# Using US Twinkling Artifact to Identify Breast Biopsy Markers: Brief Report

**DOI:** 10.1148/rycan.220168

**Published:** 2023-06-16

**Authors:** Christine U. Lee, Mara A. Piltin, Dan Moldoveanu, Matthew W. Urban, Gina K. Hesley

**Affiliations:** From the Department of Radiology, Division of Breast Imaging and Intervention (C.U.L., G.K.H.), Department of Surgery, Division of Breast and Melanoma Surgical Oncology (M.A.P., D.M.), Department of Radiology, Division of Radiology Research (M.W.U.), and Department of Physiology and Biomedical Engineering (M.W.U.), Mayo Clinic, 200 First St SW, Rochester, MN 55905.

**Keywords:** Breast, Ultrasound, Color Doppler US, Lymphatic, Artifacts, Biopsy Marker

## Abstract

Breast biopsy markers play an essential role in the surgical management of patients with clinically node-positive breast cancer. Marking a pathology-proven lymph node ensures accurate imaging assessment of response to neoadjuvant systemic therapy and decreased false-negative rates in sentinel lymph node biopsy. There is a clinically unmet need to make breast biopsy markers, particularly in the axilla, more sonographically visible or identifiable for preoperative localization purposes. Previously described color Doppler US twinkling artifact of some breast biopsy markers in in vitro gel phantoms and in ex vivo cadaveric breasts suggests that twinkling of such markers can be leveraged for improved in vivo detection. In this retrospective case series of eight female patients (mean age, 58.6 years ± 12.3 [SD]), conventional B-mode US imaging failed to identify the biopsy marker associated with a surgical target in the breast or in an axillary lymph node. However, in each patient, the marker was successfully identified with the help of color Doppler US twinkling.

**Keywords:** Breast, Ultrasound, Color Doppler US, Lymphatic, Artifacts, Biopsy Marker

Published under a CC BY 4.0 license.

See also the commentary by Whitman in this issue.

**Summary**

Readily available color Doppler US depicted the twinkling signatures of B-mode-occult Q-shaped and cork-shaped breast biopsy markers for in vivo identification.

**Key Points**

■ The 9L linear array transducer was preoperatively able to identify the twinkling signatures of five axillary Q-shaped, one breast Q-shaped, and two breast cork-shaped markers, none of which were identified with conventional B-mode US.
■ The C1–6 curvilinear array transducer provides a helpful way to survey the field for twinkling.
■ In challenging localization cases, intraoperative US coupled with a localization technique at the site of twinkling can be performed to enhance the accuracy of targeted axillary dissection.

## Introduction

There is a clinically unmet need to make breast biopsy markers more sonographically conspicuous for localization purposes (1–3). In the setting of favorable response to neoadjuvant systemic therapy, biopsy markers can be challenging to localize, with studies showing up to 24% of patients exhibiting sonographically occult markers at the time of preoperative localization (4,5). In a recent study, Tumark (Hologic) and Hydromark (Devicor Medical Products) markers, believed to improve US visualization at the time of localization, were successfully visualized and localized in only 50% (25 of 50) of patients (3). When B-mode US localization is not feasible, mammographic grid localization can be attempted; however, mammographic approaches, particularly in the axilla, can be technically challenging and uncomfortable for the patient. Occasionally, CT-guided localization is performed (6,7).

First described more than 25 years ago, the twinkling artifact at color Doppler US (8) remains incompletely understood. The literature describes twinkling of some soft-tissue markers (9,10). Data in an ex vivo study also support that actionable twinkling can be achieved, allowing for confident localization of some markers, such as the Tumark Q, Tumark Flex (which is MRI safe), and TriMark cork (Hologic) (11). In that study, which evaluated 35 commercial biopsy markers, confident twinkling was associated with a lower frequency transducer, such as the linear array 9L and the curvilinear array C1–6 on a GE Logiq E9 scanner (GE Healthcare), and lower color transmit frequencies (3–4 MHz).

If specific markers can be reliably visualized by their twinkling signatures at color Doppler US, then these markers may be indicated in cases of neoadjuvant systemic therapy with posterior breast or axillary locations where mammographic-guided localization may be prohibitive. This brief report describes the feasibility of identifying Q-shaped and cork-shaped markers by their twinkling signatures in the breast and axillary lymph nodes.

## Materials and Methods

### Patients and US Imaging

Institutional review board approval and waiver for informed consent were obtained for this single-institution retrospective case series. Eight cases, including one intraoperative case, demonstrate how US twinkling facilitated the detection of six Q-shaped markers (Tumark Professional Q; Hologic) and two cork-shaped markers (TriMark Cork; Hologic) (hereafter, Q markers and cork markers) in the breast and axilla initially found to be sonographically undetectable (Table 1). All eight patients were women with a mean age of 58.6 years ± 12.3 (SD) (range, 43–73 years). All US images were acquired with a GE Logiq E9 scanner (GE Healthcare). The time between marker placement and preoperative US, whether for localization or diagnostic scanning, ranged from 14 to 275 days.

**Table 1:**
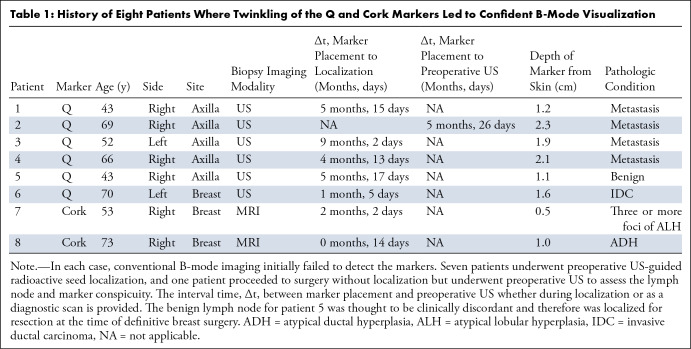
History of Eight Patients Where Twinkling of the Q and Cork Markers Led to Confident B-Mode Visualization

## Results

Preoperative, conventional B-mode imaging initially failed to identify the marker in all eight patients. In each patient, the Q and cork markers were successfully identified by their twinkling signatures on color Doppler US images using the 9L linear array transducer and the C1–6 curvilinear array transducer (Table 2). Once detected by twinkling, B-mode features of each marker were immediately confirmed with either the 9L or the ML6–15 transducers.

**Table 2:**
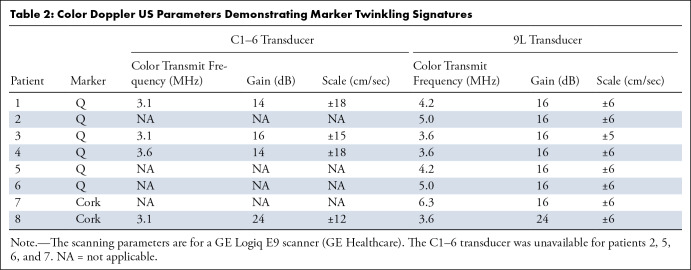
Color Doppler US Parameters Demonstrating Marker Twinkling Signatures

### Twinkling Q Markers: Five in the Axilla

Patients 1–5 had biopsy-proven invasive ductal carcinoma of the breast. Patients 1–4 had pathology-proven metastatic nodal disease, and patient 5 had a radiologically suspicious axillary node with benign cytology result from fine-needle aspiration, which was thought to be discordant. A Q marker, placed in each sampled node was on average, 1.7 cm deep from the skin, ranging from 1.1 to 2.3 cm deep. Each patient responded favorably to neoadjuvant systemic therapy.

**Figure 1:**
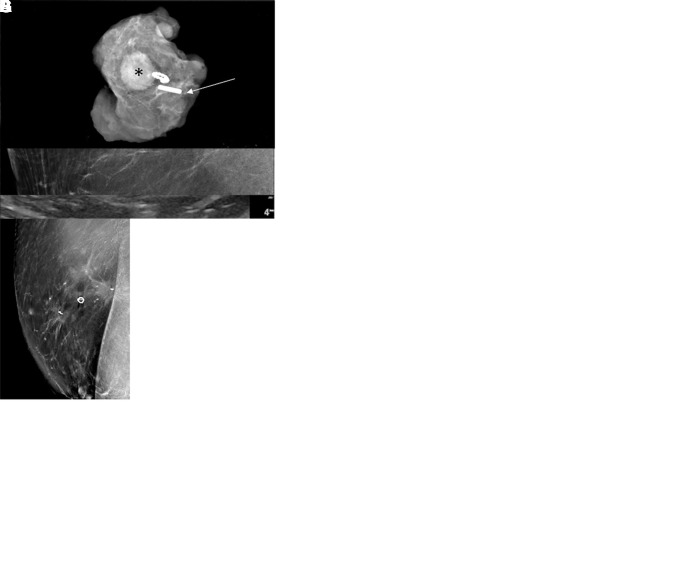
US twinkling to guide detection of the Q marker in an axillary lymph node after neoadjuvant systemic therapy. In patient 4, a 66-year-old female patient with invasive ductal carcinoma and axillary lymph node metastasis, **(A)** the postclip mammogram shows the Q marker in the sampled lymph node (arrow). The patient responded favorably to neoadjuvant systemic therapy, and at preoperative US in anticipation of radioactive seed localization (RSL) of the marked node, the Q marker could not be identified. **(B)** Survey US imaging with the C1–6 transducer identified twinkling (arrow) and its associated comet tail (chevron). **(C)** The twinkling was quickly reproduced with the 9L transducer (arrow) and **(D)** immediate B-mode interrogation confirmed features consistent with the Q marker (arrow). **(E)** The Q marker features could be reproduced, albeit more subtly, using the ML6–15 transducer (arrow). Using this information, US-guided RSL was performed, with **(F)** the postlocalization mammogram from mediolateral oblique view confirming the seed (arrow) partially in the lymph node and just inferior to the Q marker. **(G)** Specimen radiograph confirms the seed location (arrow) adjacent to the Q marker near the lymph node (black *).

**Figure 2:**
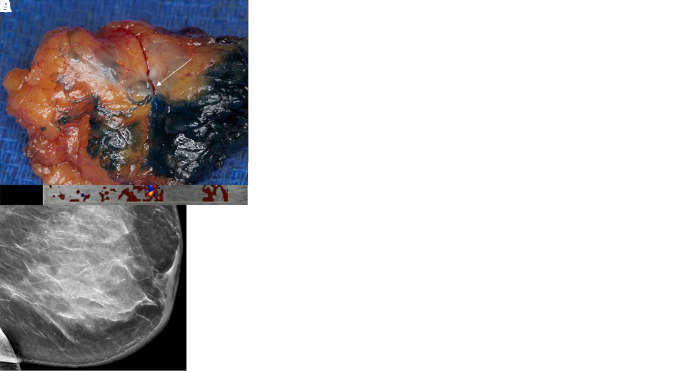
Intraoperative twinkling detection of the Q marker. In patient 3, a 52-year-old female patient with invasive ductal carcinoma and axillary lymph node metastasis, **(A)** the postclip mammogram shows the Q marker in the sampled lymph node (arrow). During preoperative routine diagnostic US, the Q marker was not identified. Intraoperative US before surgical prepping was performed the next day. **(B)** With the C1–6 transducer, a twinkling signature was identified (arrow) and **(C)** confirmed with the 9L transducer (arrow). Immediate B-mode interrogation could not confirm features of the Q marker. **(D)** To ensure that the twinkling was definitely from the Q marker, a small amount (approximately 0.5 mL) of methylene blue 0.5% (ProvayBlue 5 mg/mL, CENEXI, American Regent) was delivered by percutaneous angiocatheter under US guidance (arrow) to stain the perinodal tissue of the Q-marked lymph node and simultaneously aspirated to not create extensive tissue staining. **(E)** Once the lymph node with perinodal staining was retrieved, a specimen radiograph and pathologic evaluation confirmed the presence of the Q marker (arrow) within the specimen.

US survey for twinkling using the 9L or the C1–6 transducers (when available) allowed for confident identification of the markers, which was confirmed with immediate B-mode interrogation at the site of twinkling. Of the five patients with Q markers in the axillary lymph nodes, three underwent successful US-guided localization (iodine 125 radioactive seed is used at our institution) in anticipation of targeted axillary dissection (Fig 1), and one patient went straight to surgery, where the marked node was palpable and resected. In patient 3, the Q marker implanted more than 9 months earlier was not seen at conventional US for radioactive seed localization (RSL) the day before surgery. At the time of surgery, the authors of this publication were able to identify intraoperative twinkling characteristics prior to incision; therefore, the marked lymph node was localized intraoperatively under US guidance (Fig 2). A specimen radiograph and pathologic evaluation confirmed the presence of the Q marker in the specimen.

### Twinkling Q Markers: One in the Breast

**Figure 3:**
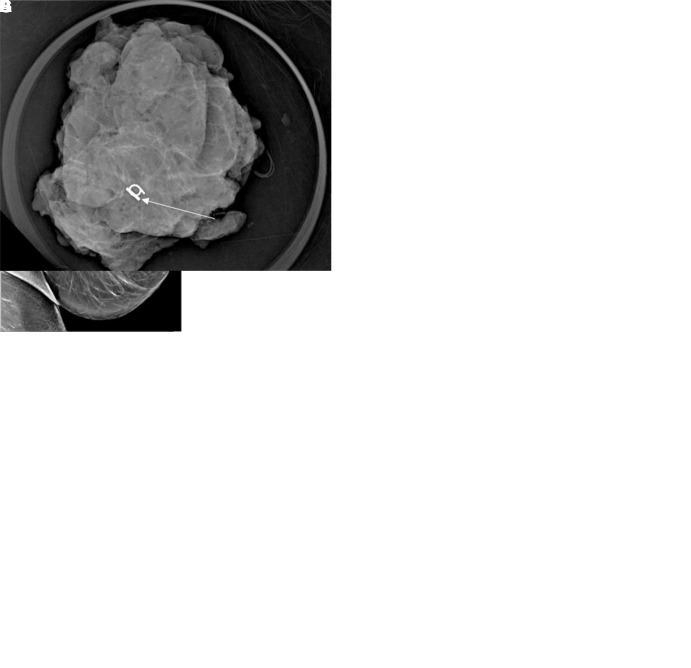
Twinkling detection of the Q marker in the breast. In patient 6, a 70-year-old female patient with invasive ductal carcinoma, **(A)** the postclip mammograms show the Q marker in the pathology-proven malignancy on the craniocaudal view (arrow) and **(B)** the mediolateral oblique view (arrow). Mammographic-guided radioactive seed localization (RSL) would have been challenging given the far posterior and inferior location of the malignancy. **(C)** Survey US imaging with the 9L transducer quickly identified twinkling (arrow), which **(D)** upon immediate interrogation with B-mode imaging, confirmed features consistent with the Q marker (arrow). US-guided RSL was performed, and **(E)** postlocalization mammograms confirmed accurate targeting on the craniocaudal view (arrow) and **(F)** the mediolateral oblique view (arrow). **(G)** Specimen radiograph confirms the seed (arrow) adjacent to the Q marker.

Patient 6 had biopsy-proven invasive ductal carcinoma marked with a Q marker in the lower left breast at far-posterior depth. Mammographic-guided RSL would have been challenging given the location of the Q-marked mass, so US-guided RSL was performed instead (Fig 3).

### Twinkling Cork Markers: Two in the Breast

Patients 7 and 8 underwent MRI-guided core needle biopsies, which showed high-risk pathologic conditions of multifocal atypical lobular hyperplasia and atypical ductal hyperplasia, respectively. The cork marker at each biopsy site twinkled, facilitating US-guided preoperative RSL. In patient 7, the cork marker was very posterior in a small-breasted patient and could not be seen mammographically for RSL. B-mode US was inconclusive, but the cork marker was easily identified by its twinkling signature using the 9L transducer, clarifying the B-mode features.

**Figure 4:**
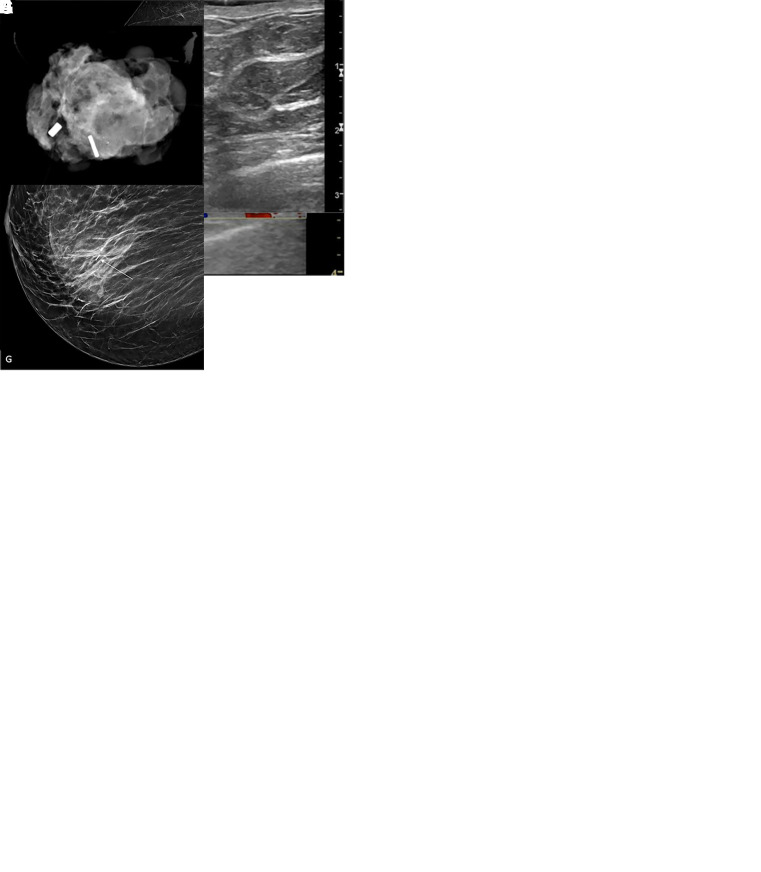
Twinkling of the cork marker. In patient 8, a 73-year-old female patient with atypical ductal hyperplasia, **(A)** the postclip mammograms show the cork marker on the craniocaudal (arrow) and **(B)** the mediolateral oblique (arrow) views. US-guided radioactive seed localization (RSL) was attempted for patient comfort. The cork marker could not be identified using conventional B-mode imaging with the ML6–15 transducer. **(C)** The C1–6 transducer quickly demonstrated the twinkling signature (arrow) which **(D)** was reproduced, albeit with less twinkling, with the 9L transducer (arrow). **(E)** Immediate repeat B-mode interrogation with an ML6–15 at the twinkling site confirmed features suggestive of the cork marker (arrow). RSL was performed under US guidance and **(F)** postlocalization mammograms confirmed the seed on the craniocaudal view (arrow) and **(G)** the mediolateral oblique view (arrow) adjacent to the cork marker. **(H)** Specimen radiograph shows the seed (arrow) adjacent to the cork marker.

In patient 8, US-guided RSL of the cork marker was attempted for patient comfort (Fig 4). Not readily detectable by using B-mode imaging, the cork marker was identified by its twinkling signature using both a C1–6 transducer and a 9L transducer. Further interrogation with an ML6–15 transducer at this twinkling location provided supportive B-mode features of the cork marker approximately 1 cm deep to the skin. RSL was successfully performed using the ML6–15 transducer.

### Scanning Parameters

With the 9L transducer, the median color US transmit frequency was 4.2 MHz from an available range of 3.1 to 6.3 MHz; the median color gain was 16 dB from a range of -20.0 to 30.0 dB; and the median color scale was ±6 cm/sec from a range of ±1 to ±100 cm/sec. For the four patients where the C1–6 transducer was available, the median color US transmit frequency was 3.1 MHz from an available range of 1.7 to 3.6 MHz; the median color gain was 15 dB from a range of -20.0 to 30.0 dB; and the median color scale was ±17 cm/sec from a range of ±1 to ±65 cm/sec.

## Discussion

To our knowledge, this brief report describes the first in-human use of US twinkling to identify the Q and cork biopsy markers in both the breast and the axillary lymph nodes. The findings presented here suggest that in cases where sonographers or radiologists cannot find the Q or the cork marker using conventional B-mode imaging for preoperative localization, marker twinkling at color Doppler US can serve as a quick, low-effort, potentially high-yield initial survey. Subsequent interrogation of the area of twinkling with B-mode imaging successfully identified markers in patients who otherwise would have had to be scheduled for CT-guided localization, potentially delaying surgery.

In all eight patients, marker twinkling was identified with the 9L transducer. When available, the C1–6 transducer readily detected twinkling of the Q and cork markers. While the 9L transducer can initially be used to identify marker twinkling, having a C1–6 transducer available could be helpful in challenging situations where relatively quick surveys of a larger area, as in the intraoperative case, are needed. Scans with the C1–6 were followed by the 9L for confirmation and better anatomic detail. While twinkling can be detected with the ML6–15 transducer, which meets transducer center frequencies of at least 12 MHz required for breast US certification by the American College of Radiology, the twinkling for some markers is not robust enough compared with twinkling using the 9L or the C1–6 transducers to allow for confident localization (11).

Marker twinkling required very few adjustments to specific scanning parameters, consistent with what is described in ex vivo studies (11,12). The lower frequency 9L and C1–6 transducers readily showed twinkling with more robust comet tails. While lower color US frequencies favored twinkling detection of the markers, it remains to be determined if lower color US frequencies are required. One report demonstrated that robust marker twinkling is insensitive to changes in color transmit frequencies (11). There is some variation in the frequency ranges that can be used for successful twinkling detection of a given marker. However, the best twinkling occurs over a range of 3–6 MHz. It may be advisable to start at the lower end of the range for a preliminary scan and refine the transmitted Doppler US frequency after that.

While most commercial biopsy markers are made of metal or generally hyperechoic materials, their small US scattering cross-sections may inhibit their visibility in complicated scattering tissue like the breast or the axilla, especially if not encountering the object at a favorable angle for obtaining strong reflectivity. Larger markers, such as the Tumark series that take advantage of the temperature-dependent expansion of nitinol, are not consistently readily visible on B-mode US images (1,3,4). The cork, a smaller (1.4 × 1.4 × 3.0 mm) marker, is challenging to detect on B-mode US images, but it exhibits an appreciable twinkling signature.

The cause of twinkling is a topic of active investigation, and surface roughness and microbubbles have been described as possible factors contributing to the twinkling signature (13,14). Surface roughness of markers corresponding to more actionable twinkling signatures (11) seems to be independent of the composition of the tested markers without embedding material. A recent technical report described a pilot study of a twinkle marker that is under development (15). Specific comparison of twinkling versus B-mode signatures of markers remains to be performed.

While all scans were performed with a GE Logiq E9 scanner, twinkling is reasonably vendor agnostic (at least for kidney stones) (8,16), as almost all scanners have color Doppler US. Consequently, these results can likely be reproduced widely in a breast imaging or surgical practice. The cases in this brief report were biased toward twinkling detection by a breast radiologist (two fellowship-trained radiologists in breast imaging and two in cross-sectional imaging); however, breast sonographers at our institution with experience ranging from 2 to 28 years have successfully used twinkling to identify B-mode occult markers. For the challenging intraoperative case in this brief report, the radiologist (C.U.L.) and the surgeon (M.A.P.) scanned the patient; while the surgeon performed the procedure, the radiologist operated the US console.

Because the cork marker deploys from the side, the marker was first deployed on the procedural tray and then subsequently deployed from a 15-gauge introducer for patient 6. The axillary Q markers were, on average, almost twice as deep from the skin compared with the breast cork markers. During the intraoperative case, US examination of the Q marker was performed prior to the skin incision. While intraoperative localization with preincision perinodal staining is not currently standard of care, it is a viable and safe technique for surgeons to label the lymph node of interest (17,18).

Calcifications can be a source of false-positive twinkling (19,20). Breasts with diffuse calcifications, such as diffuse secretory or extensive dystrophic calcifications, may limit identification of markers by their twinkling signatures; however, mammography can be helpful to differentiate between them. Other sources of twinkling include marker embedding material and air (11). Doppler blood flow could be misconstrued as twinkling, although markers with twinkling signatures generally have robust comet tails. Breast radiologists and sonographers will need to exercise the same level of care for distinguishing false-positive twinkling as they do for B-mode imaging.

In conclusion, US twinkling is largely underused in breast radiology and is likely new for many breast sonographers. The cases presented demonstrate the relative ease of using the twinkling signature of the Q and cork biopsy markers to aid in their B-mode detection in patients. This brief report highlights the advantages of twinkling to offer breast imagers and breast sonographers a straightforward, readily available, and worthwhile method to quickly detect the Q and cork markers. Future studies are needed to assess the performance of US twinkling to detect breast biopsy markers in the axillary nodes and in the breast.
